# Second Tier Testing to Reduce the Number of Non-actionable Secondary Findings and False-Positive Referrals in Newborn Screening for Severe Combined Immunodeficiency

**DOI:** 10.1007/s10875-021-01107-2

**Published:** 2021-08-09

**Authors:** Maartje Blom, Ingrid Pico-Knijnenburg, Sandra Imholz, Lotte Vissers, Janika Schulze, Jeannette Werner, Robbert Bredius, Mirjam van der Burg

**Affiliations:** 1 Department of Pediatrics, Laboratory for Pediatric Immunology, Willem-Alexander Children’s Hospital, Leiden University Medical Center, Albinusdreef 2, 2333 ZA Leiden, the Netherlands; 2 Centre for Health Protection, National Institute for Public Health and the Environment, Bilthoven, the Netherlands; 3 Department of Research and Development, Epimune GmbH, Belin, Germany; 4 Department of Pediatrics, Willem-Alexander Children’s Hospital, Leiden University Medical Center, Leiden, the Netherlands

**Keywords:** TRECs, Newborn screening, Severe combined immunodeficiency, SCID, Inborn errors of immunity, Second tier, Epigenetic immune cell counting, Next-generation sequencing, NGS

## Abstract

**Purpose:**

Newborn screening (NBS) for severe combined immunodeficiency (SCID) is based on the detection of T-cell receptor excision circles (TRECs). TRECs are a sensitive biomarker for T-cell lymphopenia, but not specific for SCID. This creates a palette of secondary findings associated with low T-cells that require follow-up and treatment or are non-actionable. The high rate of (non-actionable) secondary findings and false-positive referrals raises questions about the harm-benefit-ratio of SCID screening, as referrals are associated with high emotional impact and anxiety for parents.

**Methods:**

An alternative quantitative TREC PCR with different primers was performed on NBS cards of referred newborns (*N* = 56) and epigenetic immune cell counting was used as for relative quantification of CD3 + T-cells (*N* = 59). Retrospective data was used to determine the reduction in referrals with a lower TREC cutoff value or an adjusted screening algorithm.

**Results:**

When analyzed with a second PCR with different primers, 45% of the referrals (25/56) had TREC levels above cutoff, including four false-positive cases in which two SNPs were identified. With epigenetic qPCR, 41% (24/59) of the referrals were within the range of the relative CD3 + T-cell counts of the healthy controls. Lowering the TREC cutoff value or adjusting the screening algorithm led to lower referral rates but did not prevent all false-positive referrals.

**Conclusions:**

Second tier tests and adjustments of cutoff values or screening algorithms all have the potential to reduce the number of non-actionable secondary findings in NBS for SCID, although second tier tests are more effective in preventing false-positive referrals.

**Supplementary Information:**

The online version contains supplementary material available at 10.1007/s10875-021-01107-2.

## Introduction

Newborn screening (NBS) for severe combined immunodeficiency (SCID), the most profound form of inborn errors of immunity (IEI), improves outcomes for patients by preventing severe infections and early death. SCID is characterized by severe T-cell lymphopenia that is variably associated with an abnormal development of B- and/or natural killer (NK)-cells [1]. Patients with SCID are usually born asymptomatic but develop life-threatening infections in the first months of life [2]. Early detection by NBS enables prompt immune-restoring therapy such as hematopoietic stem cell transplantation (HSCT) or in selected case gene therapy before infections have occurred [3–5]. An increasing number of countries are adopting the T-cell receptor excision circle (TREC) assay into their screening programs to identify newborns with SCID in a pre-symptomatic phase [6–11]. TRECs are circular excised fragments of DNA formed during the T-cell receptor gene rearrangement. The δRec-φJα TREC is formed as a byproduct in approximately 70% of developing T-lymphocytes that express αβ and can therefore serve as a marker for thymic output [6]. The number of TREC copies is an indicator of thymic production of naïve T-cells as TRECs are stable and do not replicate during mitosis. TRECs can be detected in dried blood spots (DBS) by quantitative amplification using primers flanking across the joint of the circle [6, 12]. Absent or low levels of TRECs indicate reduced levels of newly formed T-lymphocytes regardless of the underlying cause.

TRECs are a highly sensitive biomarker for T-cell lymphopenia, but a non-specific marker for the primary target disease SCID, introducing the field of NBS to a palette of neonatal conditions and disorders associated with low T-cells around birth [13]. Low or absent TRECs can be identified in newborns with T-cell impairment syndromes such as Down syndrome, DiGeorge syndrome, or ataxia telangiectasia. In addition, newborns with T-cell impairment secondary to other neonatal conditions such as cardiac/gastrointestinal anomalies, chylothorax/hydrops or maternal immunosuppressant use, patients with idiopathic T-cell lymphopenia, or preterm children can have low TREC levels at birth. Finally, NBS for SCID can result in false-positive referrals; newborns with normal number of naïve T-cells as determined by flow cytometric analysis and no clinical explanation for the low TREC levels [14, 15]. The diagnosis of SCID can only be made after follow-up diagnostics including immunophenotyping and confirmatory genetic testing.

All countries that have implemented NBS for SCID are struggling with a low positive predictive value of SCID screening; the number of identified SCID patients is relatively low compared to the high number of other disorders with low TREC levels (secondary findings) [7, 10, 11, 14, 16–18]. This high number of secondary findings is met with hesitations by policymakers involved in implementation of SCID in NBS programs. A distinction can be made between *actionable* secondary findings where treatment or prevention achieves substantial health gain for the child and *non-actionable* secondary findings that may be relevant prognostically, but for which no treatment options are available or treatment options have no significant impact on outcomes [19]. Reporting actionable T-cell lymphopenia in which children will benefit from antibiotic prophylaxis, endorsing protective measures or by not receiving life-attenuated vaccines, is undisputed in the field of neonatal screening [20, 21]. However, non-actionable secondary findings and false-positive referrals raise questions about the harm to benefit ratio of screening and NBS programs should make an effort to prevent referral of these cases. High referral rates can be associated with a high work load for downstream referral centers and high diagnostic costs. More importantly, referral procedures are associated with high emotional impact for parents [17]. Even parents with a confirmed healthy newborn after follow-up can perceive their newborn as more “vulnerable” implying some effect of the referral procedure with the associated feelings of anxiety [22, 23]. There is an urgent need to reduce the number of non-actionable secondary findings and false-positive referrals in NBS for SCID for all countries that have implemented SCID screening.

Several NBS programs have tried to find the appropriate screening strategy that balances a high sensitivity, avoiding missing SCID patients, while preventing high referral rates (ranging from 0.01 to 0.09%) [7, 10, 11, 14, 16–18]. Lowering cutoff values, requesting second NBS cards, or adjusting screening algorithms for preterms could reduce the number of referrals without the need of introducing a second tier test [11, 14, 24–30]. Other programs have chosen to include a second tier test after initial TREC analysis such as next-generation sequencing (NGS) with gene panels [31, 32]. This study explores other options of second tier testing after TREC analysis such as PCR with different TREC primers and epigenetic immune cell counting in order to reduce the number of non-actionable secondary findings and false positives. Performing a PCR with primers at different positions as a second tier could prevent false-positive referrals caused by TREC region variations leading to primer/probe annealing problems [33]. Epigenetic immune cell counting as a second tier allows the measurement of relative (epi) CD3 + T-cell counts serving as more direct marker for absolute T-cells [34]. Finally, retrospective data will be used to determine whether the number of (non-actionable) secondary findings and false positives could have been reduced with a lower cutoff value or an adjusted screening algorithm. By exploring these different options, this study will make an effort to reduce the number of non-actionable secondary findings and false-positive referrals that are associated with NBS for SCID.

## Methods

### Study Population

The SONNET study (Dutch implementation pilot) screened 201,470 newborns for SCID from April 2018 to December 2020 [17]. NBS cards of these newborns were included in this study. The SONNET study was approved by the Medical Ethics Committee of the Erasmus MC, University Medical Center, Rotterdam (MEC-2017–1146).

NBS cards of newborns with low TRECs (*N* = 56) and anonymized healthy controls (*N* = 80) were analyzed with a second PCR with different TREC primers. Epigenetic immune cell counting was performed on NBS cards of anonymized healthy controls (*N* = 331) and newborns with low TRECs (*N* = 59). DNA was isolated for TREC region sequencing from NBS cards of healthy controls (*N* = 12), idiopathic T-cell lymphopenia cases (*N* = 4), and false-positive referrals (*N* = 8). Some NBS cards of referred newborns were excluded for second tier analysis due to insufficient DBS material or parental objection to anonymized scientific research with NBS cards. The use of NBS cards was approved by the National Institute for Public Health and the Environment (RIVM; no 2019–3).

### TREC Measurements

Initial TREC measurements were performed with the SPOT-it™ Neonatal Screening kit (ImmunoIVD, Stockholm, Sweden) according to manufacturer’s instructions and a preset screening algorithm [17]. As a second tier option, TREC levels were measured with the NeoMDx TREC/KREC/SMN1 multiplex assay (PerkinElmer, Turku, Finland) according to the manufacturer’s instructions. RRP30 was used as internal control. NBS samples were punched in a 96-well plate; after which, wash solution and elution solution were added in turn before different incubation steps. After DNA extraction, 3 μL of DNA was added to 12 μL of master mix. The PCR plate was sealed and analyzed on a QuantStudio 5 qPCR system.

### Epigenetic Immune Cell Counting

Epigenetic immune cell counting was performed by amplification of cell-type-specific demethylated genomic regions according to the protocol of the manufacturer (Epimune GmbH, Berlin, Germany). In short, DNA was extracted from three 3.2-mm blood punches by adding 68 μL lysis buffer and 11 μL of proteinase K followed by lysis at 56 °C for 15 min with 900-rpm shaking (ThermoMixer C, Eppendorf, Hamburg, Germany). Ammonium bisulfite (180 μL) and tetrahydrofurfuryl alcohol (TFHA; 60 μL) were added followed by incubation for 45 min at 80 °C; after which, binding buffer (580 μL) and isopropanol (380 μL) were added. Punches were removed by transferring the mixture into a fresh 2-mL tube, and magnetic beads (MagBind Particles HDQ) were added for DNA binding. After two extensive washing steps and a drying step at 65 °C for 10–15 min without shaking, 40 μL of elution buffer was added. The samples were incubated at 65 °C for 7–10 min at 1400 rpm; after which, the eluate was transferred into fresh 0.2-mL tubes. Converted DNA was stored at – 20 °C. For qPCR, 1.5 μL of the DNA was pipetted into a 384-well plate in triplicate, followed by 3.5 μL of the CD3 + and glyceraldehyde-3-phosphate dehydrogenase (GAPDH)–specific primer/probe master-mix. The plate was sealed and analyzed using the QuantStudio 6 Flex qPCR system (Thermo Fisher, Waltham, MA, USA). Relative (epi) CD3 + T-cell counts (% of CD3 demethylated copies of GAPDH demethylated copies) were calculated as previously described [34].

### TREC Sequencing

Based on the primer/probe flanking regions provided by the manufacturer (ImmunoIVD), new primers up- and downstream of the flanking regions were designed (TREC forward: [GGCAAAATGGGGCTCCTG]; TREC reverse [GACATTTGCTCCGTGGTCTG])*.* DNA was extracted from three 3.2-mm punches using the GenElute Mammalian Genomic DNA Miniprep kit (SIGMA-Aldrich, Saint Louis, MO, USA) with an adapted protocol. Isolated DNA was mixed with 2.5 μL 10 × Gold Buffer, 1.5 μL 25 mM MgCl, 0.25 µL 20 mM dNTPs, 0.5 μL 10 mM primers, and 0.1 μL polymerase Ampli TagGOLD (5 U/µL). PCR amplification was done by a hot start, followed by 40 cycles of 30″ 94 °C denaturation step, 45″ 63 °C primer annealing, and 1′ 30″ 72 °C elongation step, finished with 10′ 72 °C. Sanger sequencing was facilitated by the Leiden Genome Technology Center (LGTC).

### Adjustment of the TREC Cutoff Value and Screening Algorithm

Based on retrospective data of the SONNET study [17], the number of secondary findings and false-positive referrals were determined with a different cutoff value for the initial TREC measurements (TREC ≤ 6 copies/punch instead of TREC ≤ 10 copies/punch with SPOT-it assay) and with an adjusted screening algorithm. As part of the follow-up plan, peripheral blood of referred newborns used for flow cytometry (7 to 10 days after birth) was spotted on filter paper and reanalyzed with the SPOT-it TREC assay. Based on this data, it was determined which newborns would be directly referred (urgent TREC-positive cases with TRECs 0–2 copies/punch) and which newborns would have been referred after a second NBS card (newborns with TRECs 2–10 copies/punch) with a new screening algorithm.

### Statistical Analysis

Descriptive statistics were used to summarize the distribution of TREC levels and relative (epi) CD3 + T-cell counts. For correlation analysis, Pearson *r* correlation tests were used, while unpaired *t*-tests were used for group comparison. Epigenetic CD3/GAPDH copies were log-transformed and used to estimate a normal distribution with 99.9% confidence interval. *P*-values < 0.05 were considered statistically significant. All *P*-values are two-sided. Statistical analysis was carried out with SPSS version 25.0 for Windows (SPSS, Inc., Chicago, IL, USA).

## Results

### Results SONNET Study

In total, 62 out of 201,470 newborns were identified in the SONNET study with low TREC levels (April 2018 to December 2020). These newborns were referred for follow-up diagnostics, leading to a referral rate of 0.03%. One X-linked SCID patient was identified with absent TRECs and absent T-cells [17]. In the other 61 newborns, eight newborns had normal flow cytometry results without a known underlying cause for the low TREC levels (false positives). There were 53 newborns with non-SCID T-cell lymphopenia of which the diagnoses are specified in Table S1.

### SNPs Identified in the TREC Region of False-Positive Referrals

**Fig. 1:**
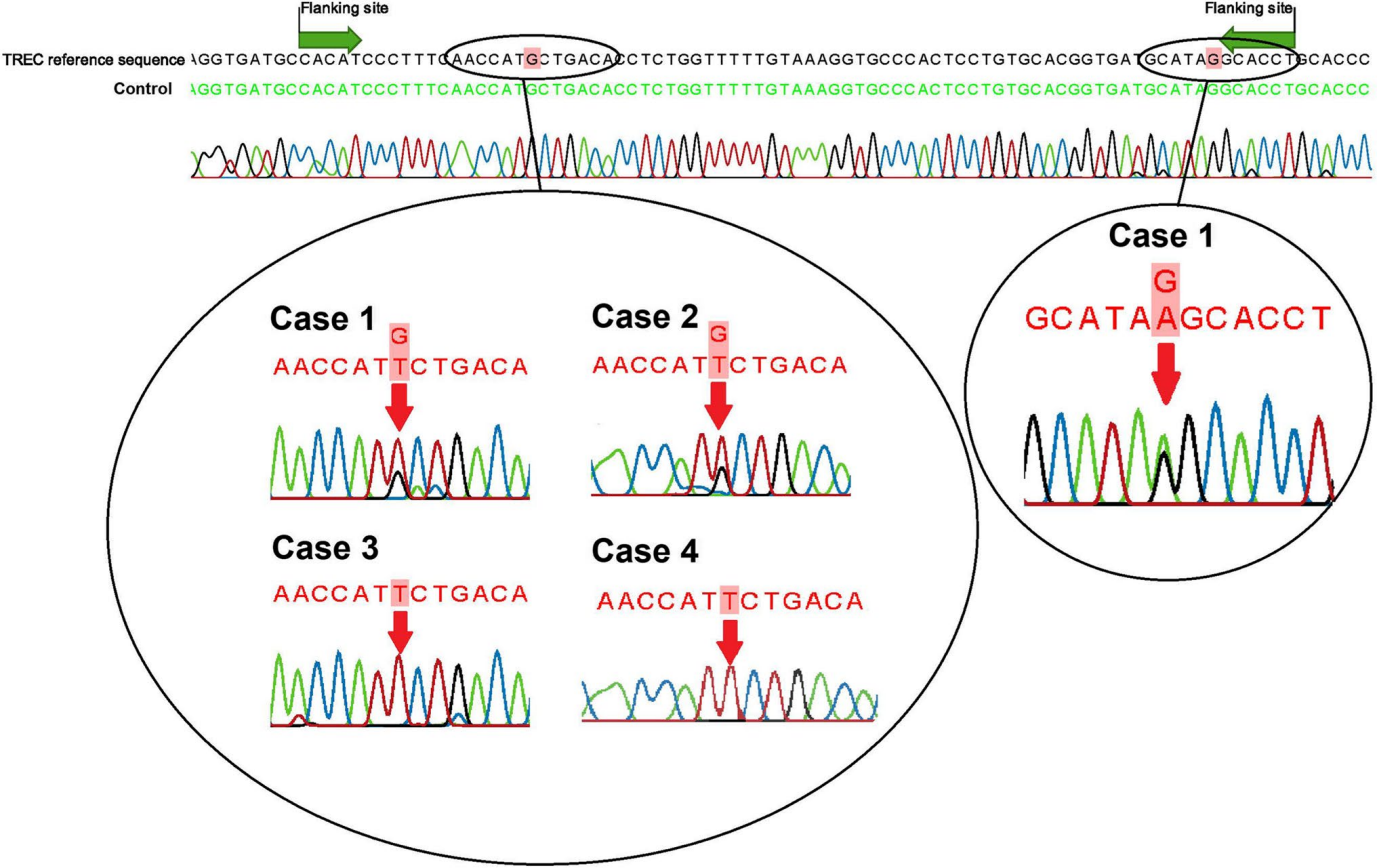
Sequence analysis of TREC region of false-positive referrals with normal flow cytometry and low/undetectable TREC levels in peripheral blood used for flow cytometry spotted on filter paper (*N* = 4). The flanking regions provided by the manufacturer are depicted with green arrows. SNPs (G > T and G > A) are indicated with the red arrows

Eight of 62 (13%) referred newborns had normal flow cytometric results and no clinical explanation for the low TREC levels. As part of the follow-up plan, peripheral blood used for flow cytometry was spotted on filter paper and reanalyzed with the SPOT-it TREC assay for seven false-positive cases. Three false-positive cases had normal TREC levels in the peripheral blood DBS, as would be expected with normal absolute T-cell counts measured in the same blood sample (data not shown). However, four false-positive cases with normal immunophenotyping had low or undetectable TRECs in repeated TREC analysis on peripheral blood DBS (Table 1). Variations in the TREC region might lead to primer/probe annealing problems and therefore amplification failure in these false-positive referrals. With sequencing, SNPs were identified in the TREC region (defined by manufacturer, personal communication) of these four false-positive cases, whereas no variations were found in the false-positive cases with normalized TREC levels in peripheral blood (Fig. 1, Table 1). Case 1 had two heterozygous SNPs, whereas case 2 had one heterozygous SNP. Cases 3 and 4 had complete amplification failure of TREC and had a SNP present on both alleles (Fig. 1). The presence of a homozygous SNP might lead to a complete failure of TREC amplification, whereas the presence of two heterozygous SNP will probably lead to less efficient amplification, but not in the absence of TRECs. No variations were found in healthy control neonates (*N* = 12) and referred newborns with idiopathic T-cell lymphopenia (*N* = 4).

**Table 1:** SNPs identified in primer/probe binding sites of false-positive referrals with low/absent TREC levels

	TREC from initial NBS cards in triplicate (copies/punch)	TREC from peripheral blood card in triplicate (copies/punch)	SNP rs377686467^a^	SNP rs1466932014^b^
Case 1	2–3–6	7–3–7	G/T	G/A
Case 2	5–3–3	11–10–5	G/T	
Case 3	0–0–0	1–0–0	T/T	
Case 4	0–0–0	0–0–0	T/T	
Case 5	10–14–6	80–114–96	No SNP identified	
Case 6	5–11–10	114–41–127	No SNP identified	
Case 7	5–13–8	72–66–102	No SNP identified	
Case 8	1–3–1	Not measured	No SNP identified	

^a^dbSNP: NC_000014.9:g.22475276G > T; allele frequency of T = 0.0005 (1/2188, ALFA Project)[43]

^b^dbSNP:NC_000014.9:g.22386840G > A; allele frequency of A = 0.00008 (1/11862, ALFA Project)[44]

### PCR with Different Primers as Second Tier Test

**Fig. 2:**
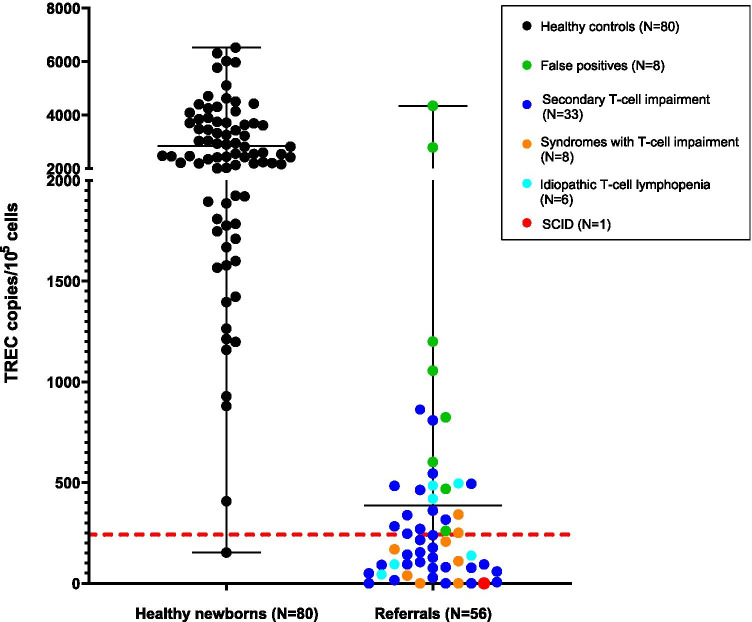
TREC levels in copies/10^5^ cells of healthy newborns (*N* = 80) and referred newborns (*N* = 56) measured with the NeoMDx assay (PerkinElmer). The red dotted line is the cutoff of the manufacturer at TREC ≤ 242 copies/10^5^ cells. The black line shows the mean and range of the TREC copies/10^5^ cells. Diagnoses of referrals are categorized as SCID (red), false-positive cases (green), idiopathic T-cell lymphopenia (turquoise), syndromes with T-cell impairment (orange), and secondary T-cell impairment (blue)

Because of the presence of SNPs in the TREC region, the commercially available NeoMDx PCR with primers at other positions was used as a second tier. Referred newborns with low TREC levels (*N* = 56), including the eight false positives, and healthy newborns (*N* = 80), were analyzed. Mean TREC value of healthy controls was 2844 copies/10^5^ cells (range 152–6522 copies/10^5^ cells), whereas the mean TREC value of the referrals (*N* = 56) was 387 copies/10^5^ cells (range 0–4348 copies/10^5^ cells). Pearson *r* correlation between TREC levels measured with the SPOT-it kit assay and the NeoMDx assay was 0.74 (*P* < 0.001). Of the referred newborns, 45% of the referrals (25 out of 56) had TREC levels above the cutoff value proposed by the manufacturer (≤ 242 copies/10^5^ cells) (Fig. 2). Diagnoses below cutoff included SCID (*N* = 1), syndromes with T-cell impairment (*N* = 6), idiopathic T-cell lymphopenia (*N* = 3), and secondary T-cell impairment due to various reasons (*N* = 21) (Table S1 and Table S2). All false-positive cases had TREC levels above cutoff and in the range of the healthy controls (range 602–4348 copies/10^5^ cells). One newborn with TRECs of 19 copies/punch measured with the SPOT-it assay had TREC levels below cutoff measured with the NeoMDx assay (TREC 153 copies/10^5^ cells).

### Epigenetic Immune Cell Counting as a Second Tier Test

**Fig. 3:**
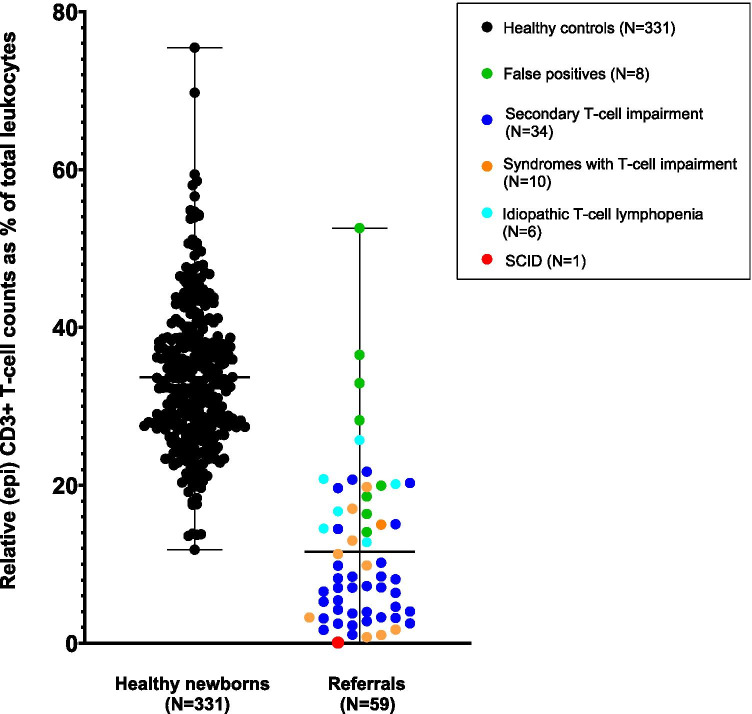
Relative (epi) CD3 + T-cell counts as a percentage of total leukocytes of healthy newborns (*N* = 331) and referred newborns (*N* = 59) measured with epigenetic qPCR (Epimune GmbH). The mean and range are depicted with a black line. Diagnoses of referrals are categorized in SCID (red), false-positive cases (green), idiopathic T-cell lymphopenia (turquoise), syndromes with T-cell impairment (orange), and secondary T-cell impairment (blue)

**Fig. 4:**
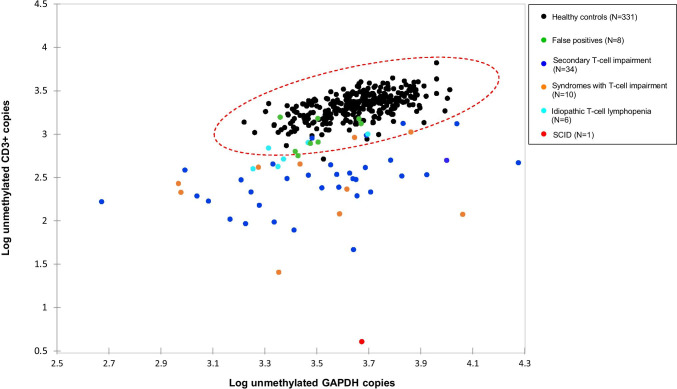
Log-transformed epigenetic CD3/GAPDH copies with a 99.9% confidence interval (red ellipse). Healthy newborns (*N* = 331) are depicted in black. Diagnoses of referrals are categorized in SCID (red), false-positive cases (green), idiopathic T-cell lymphopenia (turquoise), syndromes with T-cell impairment (orange), and secondary T-cell impairment (blue). Twenty-five percent (15 out of 59) of the referrals with low TREC levels fell within the 99.9% confidence ellipse of the healthy controls

Next, epigenetic immune cell counting was studied as second tier test. This assay is based on amplification of a T-cell-specific demethylated genomic region and measurement of relative (epi) CD3 + T-cell counts in DBS. Mean relative (epi) CD3 + T-cell count as a percentage of leukocytes (CD3%) in healthy newborns was 33.7% (*N* = 331; range 11.85–75.47%), while mean relative (epi) CD3 + T-cell count for referred newborns with low TRECs was 11.6% (*N* = 59; range 0.09–52.60%) (*P* < 0.001). Pearson *r* correlation between TRECs and unmethylated CD3 copies was 0.59 (*P* < 0.001), suggesting a moderate correlation, which implies that epigenetic qPCR can generate different results as a second tier compared to TREC analysis as a first tier. Twenty-four of 59 referrals had relative (epi) CD3 + T-cell counts in the range of healthy controls (41%), including all false-positive cases with confirmed SNPs (Fig. 3). For (epi) CD3 + T-cells and GAPDH measurements, 15 out of 59 referrals with low TREC levels (25%) fell within the 99.9% confidence interval ellipse of the healthy controls (Fig. 4). The (non)-actionable diagnoses of these potentially prevented referrals are listed in Table S1 and Table S2. Pearson *r* correlation between absolute CD3 + T-cell numbers determined with flow cytometry and TREC levels measured in peripheral blood of 36 referred newborns was 0.57 (*P* < 0.001). In contrast, a strong correlation was found (*r* = 0.86 (*P* < 0.001)) between absolute T-cell numbers and relative (epi) CD3 + T-cell counts as a percentage of total leukocytes measured with epigenetic qPCR.

### Effect of Adjustment of the TREC Cutoff Value on Number of Referrals

When applying a lower TREC cutoff value of ≤ 6 copies/punch instead of a cutoff value of TREC ≤ 10 copies/punch to the retrospective data of the referrals in the SONNET study, 37 of 62 referrals (60%) had TREC levels below 6 copies/punch (Table S1). As a variation in the TREC region of the false-positive referrals can lead to TREC amplification failure and very low or absent TREC levels, lowering the cutoff would not prevent this type of referrals.

### Effect of Adjustment of the Screening Algorithm on Number of Referrals

In the SONNET study, all children with TRECs below the cutoff of 10 were referred (*N* = 62). However, if the screening algorithm would be adjusted in such a way that only newborns with TRECs between 0 and 2 copies/punch were referred directly and newborns with TRECs between 2 and 10 copies/punch would require a second confirmation NBS card, only 14 out of 62 (23%) would have been directly referred; 48/62 (77%) would require a second NBS card. Based on retrospective analysis of DBS of peripheral blood taken on the day of the presumed confirmatory NBS card, 17 out of 32 tested newborns (53%) had a normal TREC levels (above cutoff) and would not have been referred according to this adjusted screening algorithm. Mainly, newborns with false-positive screening results without a SNP and newborns with secondary T-cell impairment that resolved in the first week after birth had normal TREC levels in peripheral blood (Table S1).

### Effect of Combined Strategies and Second Tier Tests on Number of Referrals

**Fig. 5:**
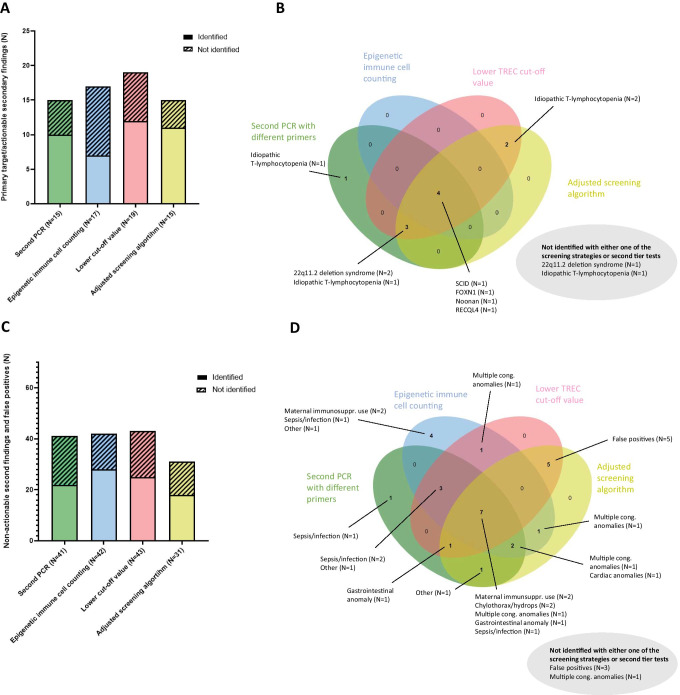
Effect of combined different screening strategies and second tier tests on number of referrals.** A** Number of target disease (SCID) and actionable secondary findings identified/not identified per strategy. Not all referrals were analyzed with each screening strategy/second tier test. **B** Overlap between the four different screening strategies in identifying/not identifying SCID and actionable secondary findings. Only actionable cases analyzed with all four screening strategies were included in the Venn diagram (*N* = 12). Two out of twelve referrals were not identified with any of the screening strategies **C** Number of non-actionable secondary findings and false-positive cases identified/not identified per strategy. Not all referrals were analyzed with each screening strategy/second tier test. **D** Overlap between the four different screening strategies in identifying and not identifying non-actionable secondary findings and false-positive cases. Only non-actionable cases analyzed with all four screening strategies were included in the Venn diagram (*N* = 30). Four out of 30 referrals were not identified with any of the screening strategies [45]

Combining data of the different screening strategies and second tier tests show both overlap and differences between the identified and not identified actionable and non-actionable secondary findings (Fig. 5). As previously mentioned, not all referrals were analyzed with all screening strategies due to insufficient DBS material or parental objection to anonymized scientific research with NBS cards (Fig. 5A, C Table S1 and Table S2). Only referrals analyzed with all four screening strategies and second tier test were included for overlap analysis (*N* = 42 out of 62 referrals) (Fig. 5B, D). All screening strategies, second tier tests, and combinations of both are able to identify patients with SCID (*N* = 1), heterozygous FOXN1 variant (*N* = 1), Noonan syndrome (*N* = 1), and RECQL4 (*N* = 1), while not identifying or “missing” one 22q11.2 deletion syndrome patient and one patient with idiopathic T-lymphocytopenia. Lowering the TREC cutoff value or adjusting the screening algorithm would result in similar numbers of (non-)actionable secondary findings. Outcomes of combined second tier tests with adjustments in cutoff value or screening algorithms would be highly dependent on chosen cutoff values or normal ranges.

## Discussion

Universal NBS for SCID was made possible by the development of a TREC assay utilizing DBS. TRECs are a highly sensitive biomarker for T-cell lymphopenia, but a non-specific marker for the primary target SCID, introducing the field of NBS to a palette of neonatal conditions and disorders associated with low T-cells around birth. NBS programs should make an effort to prevent non-actionable secondary findings and false-positive referrals that are associated with parental anxiety and costs for potentially unnecessary invasive tests. This study explored second tier test options after TREC analysis and adjustments in the TREC cutoff value and screening algorithm in order to reduce the number of referrals and emotional impact for parents and increase the positive predictive value for NBS for SCID.

Using a second PCR with different TREC primers has the potential to reduce the number of referrals, in particular the false-positive referrals with a SNP in the primer/probe region of the initial SPOT-it TREC assay. Not all laboratories will experience these primer/probe annealing problems due to SNPs, because that depends on the frequency of SNPs in the population and the chosen TREC assay or primer/probe combination. One has to keep in mind that each laboratory should determine its own cutoff values and that the referral rate will be highly depending on this chosen cutoff. This second tier test option is not limited to commercially available assays as screening laboratories can develop “in-house methods” with new primer sets. However, as some laboratories have strict criteria for accreditation and prefer using CE-IVD assays, the research-only NeoMDx assay is also available as a CE-IVD marked assay (EONIS SCID-SMA kit, PerkinElmer). Although PCR with different primers might not result in a much lower referral rate, this option does provide rapid availability of results with a feasible assay for any screening laboratory at relatively low costs.

Immunophenotyping and measuring absolute cell counts by flow cytometry is considered the golden standard in diagnostics for SCID. We showed that relative (epi) CD3 + T-cell counts measured with epigenetic qPCR had a much stronger correlation to absolute T-cell counts than TRECs (Pearson *r* correlation = 0.57 versus 0.86, *P* < 0.001), making it a more sensitive marker for T-cell lymphopenia. A potential improvement of the assay would be the identification of naïve T-cells. By including the measurement of naïve T-cells or recent thymic emigrants (RTEs), one could identify SCID cases with potentially maternal T-cell engraftment, or leaky SCID cases with oligoclonal T-cell expansion as seen in Omenn syndrome [2, 35]. A pitfall of measuring relative cell counts in contrast to absolute cell counts is that proportional cell numbers within the corresponding reference range might not accurately reflect the clinically relevant alterations in the patient. Patients could have very low numbers of total leukocytes with normal percentage of T-cells concealing a severe T-cell lymphopenia. Interestingly, epigenetic immune cell counting is not limited to measurement of CD3 + T-cells in DBS. In addition to SCID, there are many IEI that could benefit from early diagnosis and intervention if a suitable NBS test was available. With the Wilson and Jungner screening criteria in mind, some IEI might qualify for a disease that causes an important health problem and would benefit from early detection and treatment by preventing severe infections and auto-immunity [36]. With epigenetic immune cell counting, quantitative defects of other immune cell populations such as B-cells or neutrophils could offer early detection of X-linked agammaglobulinemia (XLA) and severe congenital neutropenia (SCN) shortly after birth [34]. Automating the protocol would increase the throughput time for higher workloads, making epigenetic immune cell counting a valuable addition to future NBS for IEI.

Several countries have adjusted their cutoff value after pilot studies [11, 14, 24–26] or have implemented the request of a second NBS card for newborns with low TREC levels prior to referral into their screening algorithm [10, 28, 37]. Requesting a second NBS is based on the fact that TRECs can normalize in the first week(s) after birth in newborns with secondary T-cell impairment or in false-positive referrals. Lowering the cutoff value or requesting a second NBS card will not prevent the referral of false-positive cases with a SNP leading to amplification failure. In the Netherlands, requesting a second NBS card was introduced with national implementation of NBS for SCID on 1 January 2021. A distinction was made between “TREC urgent positives” with absent/very low TREC levels and newborns with slightly higher, below cutoff TREC levels similar to the Polish/German trans-border cooperation for NBS for SCID [10]. The Israeli NBS program does not discriminate between TREC-positive cases and requests a second NBS card for all newborns with TRECs below cutoff [37]. There is no international consensus on the time frame in which this repeated sampling should be performed, but a balance between time for TRECs to normalize and the risk of developing infections in newborns with severe T-cell problems should be pursued. In the Netherlands, a second confirmation NBS card is collected after 7 days for newborns with TRECs between 2 and 10 copies/punch. In the coming years, more evidence on these screening algorithms, urgently positive TREC cutoff values and time frames for repeated sampling, will be collected. Finally, we should acknowledge that repeated sampling is not without anxiety and emotional insecurity for parents and additional distress for the newborn. Implementing repeating sampling in any screening algorithm should be well-thought-out. Clear information provision for parents is of utmost importance in this process [38, 39].

It can be challenging to make clear statements about the palette of actionable and non-actionable secondary findings, as the exact case definition of *actionable* is not specified. One could argue that the term “actionable” is mainly depending on the absolute T-cell numbers and the duration of the T-cell defect, but there is need for uniform case definitions across international NBS programs for SCID. Combining different screening strategies and second tier tests is not the preferred option for NBS programs. Multiple second tier tests would require more DBS material, which is not always available and might be required for other (more urgent) second tier NBS tests. In addition, a combination of screening strategies could introduce a significant delay in reporting the definitive screening results and referral of the newborn. The different screening strategies discussed in this paper show different numbers of identified and “missed” *actionable* secondary findings (Fig. 5, Table S1 and Table S2). From a clinical perspective, early diagnosis and management of *actionable* T-cell lymphopenia provide a clear and valuable health benefit for the child. However, NBS is aimed at detection of the primary target disease, and from a public health perspective, programs aimed at neonatal screening should try to avoid secondary findings where possible. Opting for a test method with the lowest chance of secondary findings, regardless of their actionality, is the preferred option. Each public health program should take these different perspectives into account when deciding on a balanced harm to benefit ratio for their NBS programs.

In addition to the options discussed in this study, other programs tried to reduce the number of secondary findings by implementing NGS as a second tier test [31, 32]. NGS with targeted gene panels on the initial NBS card will facilitate and accelerate final molecular diagnoses of affected newborns while providing useful information for management and follow-up. Targeted NGS has a rapid turnaround time, and a higher TREC cutoff value in combination with NGS allows for the detection of atypical and leaky SCID with clear HSCT indication [31]. However, it is important to note that if no pathogenic variants are identified with NGS, disease-causing variants could still be present in genes not included in the NGS gene panel. Moreover, structural and intronic variants can be missed with exon-based targeted NGS. A “safety net” including follow-up should be included for apparently healthy babies with low TRECs without pathogenic NGS findings. Genes included in the panels need to be constantly updated, a plan for managing “variants of unknown significance” needs to be developed, and functional validation assays are required to prove pathogenicity of novel variants [31]. NGS is associated with relatively high analyses and equipment costs and a cost-effectiveness analysis including efficiency gains and improved management could help NBS policy makers when discussing implementation of NGS [40]. The successful implementation of NGS in NBS as a second tier opens discussion for expansion of NBS for immunodeficiencies by using NGS as a first tier [41, 42]. Sequencing without including any phenotypic markers as a first tier option remains challenging due to missing links between disease pathogenesis and gene expression and the inability to distinguish underlying pathogenic variants from the high number of genomic variations [5]. Overall, high-throughput NGS analysis using targeted gene panels as a second tier after TREC analysis can reduce (false positive) referrals while increasing the diagnostic precision and the specificity in NBS programs for SCID. Before implementation, any second tier test option should be evaluated in a broader perspective taking sensitivity, specificity, costs, feasibility for screening laboratories, and throughput time into account.

In conclusion, second tier tests or adjustments in cutoff values and screening algorithms all have the potential to reduce the number of non-actionable secondary findings and false-positive referrals in NBS for SCID. A second PCR with different primers would prevent false-positive referrals caused by TREC amplification failure attributed to variations in the TREC primer/probe region. Epigenetic immune cell counting could also serve as a first tier in NBS for IEI if the protocol would be automated and throughput time increased. Rapid NGS seems to better fit the role of a second tier test, facilitating and accelerating molecular diagnoses of affected newborns. These findings will be of aid to any NBS program by attempting to prevent non-actionable secondary findings and false-positive referrals and increase the predictive value for NBS for SCID.

## Supplementary Information

Below is the link to the electronic supplementary material.

Supplementary file1 (DOCX 38.4 KB)

## Data Availability

All data generated or analyzed during this study are included in this published article and its supplementary information.
