# Supplementary material for: Second Tier Testing to Reduce the Number of Non-actionable Secondary Findings and False-Positive Referrals in Newborn Screening for Severe Combined Immunodeficiency
Source: J Clin Immunol. 2021 Aug 9;41(8):1762–73. doi: 10.1007/s10875-021-01107-2 (PMC8604867; doi:10.1007/s10875-021-01107-2)
Supplement: Supplementary file 1 — Supplementary file1 (DOCX 38.4 KB) [file 10875_2021_1107_MOESM1_ESM.docx]

**Supplemental data**

**Table S1. Sub-diagnoses of (non-)actionable secondary findings that would be identified or not identified after second tier testing or screening algorithm adjustments.**

|  | **SONNET**  **study**  **(SPOT-it assay)** | **PCR with different primers** | | | **Epigenetic immune cell counting** | | | **If a lower cut-off value was applied**  **(TREC≤6 copies/punch)** | | | **If a new screening algorithm was applied ^c^** | | |
| --- | --- | --- | --- | --- | --- | --- | --- | --- | --- | --- | --- | --- | --- |
|  | Identified  *N* | Identified  *N* | Not identified  *N* | Not tested  *N* | Identified  *N* | Not identified  *N* | Not tested  *N* | Identified  *N* | Not identified  *N* | Not included  *N* | Identified  *N* | Not identified  *N* | Not included  *N* |
| **Target disease** | | | | | | | | | | | | | |
| **SCID (X-Linked)** | 1 | 1 | 0 | 0 | 1 | 0 | 0 | 1 | 0 | 0 | 1 | 0 | 0 |
| **Actionable secondary findings** | | | | | | | | | | | | | |
| **22q11.2 deletion syndrome** | 4 | 2 | 2 | 0 | 1 | 3 | 0 | 2 | 2 | 0 | 2 | 1 | 1 |
| **Trisomy 21** | 3 | 1 | 0 | 2 | 2 | 0 | 1 | 1 | 2 | 0 | 1 | 0 | 2 |
| **Noonan syndrome** | 1 | 1 | 0 | 0 | 1 | 0 | 0 | 1 | 0 | 0 | 1 | 0 | 0 |
| **Heterozygous FOXN1 variant** | 1 | 1 | 0 | 0 | 0 | 1 | 0 | 1 | 0 | 0 | 1 | 0 | 0 |
| **RECQL4 variant** | 1 | 1 | 0 | 0 | 1 | 0 | 0 | 1 | 0 | 0 | 1 | 0 | 0 |
| **RMRP variant** | 1 | 0 | 0 | 1 | 1 | 0 | 0 | 1 | 0 | 0 | 1 | 0 | 0 |
| **Idiopathic T-cell lymphocytopenia** | 7 | 3 | 3 | 1 | 0 | 6 | 1 | 4 | 3 | 0 | 3 | 3 | 1 |
| **Non-actionable secondary findings** | | | | | | | | | | | | | |
| **Multiple congenital anomalies ^a^** | 6 | 2 | 4 | 0 | 5 | 1 | 0 | 2 | 4 | 0 | 3 | 2 | 1 |
| **Congenital diaphragmatic hernia** | 6 | 3 | 1 | 2 | 5 | 0 | 1 | 3 | 3 | 0 | 1 | 0 | 5 |
| **Cardiac anomalies** | 2 | 1 | 1 | 0 | 2 | 0 | 0 | 0 | 2 | 0 | 1 | 0 | 1 |
| **Gastrointestinal anomalies** | 2 | 2 | 0 | 0 | 1 | 1 | 0 | 2 | 0 | 0 | 2 | 0 | 0 |
| **Chylothorax and hydrops** | 3 | 3 | 0 | 0 | 3 | 0 | 0 | 3 | 0 | 0 | 2 | 0 | 1 |
| **Sepsis and severe infections** | 7 | 5 | 2 | 0 | 5 | 2 | 0 | 5 | 2 | 0 | 1 | 4 | 2 |
| **Maternal immunosuppr. use** | 4 | 2 | 2 | 0 | 4 | 0 | 0 | 2 | 2 | 0 | 2 | 2 | 0 |
| **Other neonatal conditions ^b^** | 5 | 3 | 2 | 0 | 3 | 2 | 0 | 3 | 2 | 0 | 1 | 2 | 2 |
| **False-positives** | 8 | 0 | 8 | 0 | 0 | 8 | 0 | 5 | 3 | 0 | 5 | 3 | 0 |
| **Total (N)** | **62** | **31** | **25** | **6** | **35** | **24** | **3** | **37** | **25** | **0** | **29** | **17** | **16** |

a. Multiple congenital anomalies included newborns with nemaline rod myopathy (de novo variant ACTA1), holoprosencephaly/diaphragmatic hernia due to GLI1 variant, MADD deficiency and other defects.

b. Other neonatal conditions included severe asphyxia, dysmaturity, high doses of dexamethasone and start of chemotherapeutics prior to sample collection.

c. Number of (secondary) findings identified and not identified if a new screening algorithm was applied during the SONNET-study. Only newborns with TREC 0-2 (N=14, SCID case included) and newborns with TREC measurements in peripheral blood (N=32) were included.

**Table S2. Referred newborns (N=62) that are identified (+), not identified (-) or not tested (NT) after second tier testing or screening algorithm adjustments.**

| **Patient number** | **Diagnosis** | **PCR with different primers** | **Epigenetic immune cell counting** | **If a lower cut-off value was applied**  **(TREC≤6 copies/punch)** | **If a new screening algorithm was applied** |
| --- | --- | --- | --- | --- | --- |
| 1 | SCID | + | + | + | + |
| 2 | 22q11.2 deletion syndrome | + | - | + | + |
| 3 | 22q11.2 deletion syndrome | + | - | + | + |
| 4 | 22q11.2 deletion syndrome | - | - | - | - |
| 5 | 22q11.2 deletion syndrome | - | + | - | NT |
| 6 | Trisomy 21 | + | + | + | NT |
| 7 | Trisomy 21 | NT | + | - | + |
| 8 | Trisomy 21 | NT | - | - | NT |
| 9 | Noonan syndrome | + | + | + | + |
| 10 | Heterozygous FOXN1 variant | + | + | + | + |
| 11 | RECQL4 variant | + | + | + | + |
| 12 | RMRP variant | NT | + | + | + |
| 13 | Idiopathic T-cell lymphocytopenia | - | - | + | + |
| 14 | Idiopathic T-cell lymphocytopenia | - | - | + | + |
| 15 | Idiopathic T-cell lymphocytopenia | + | - | + | NT |
| 16 | Idiopathic T-cell lymphocytopenia | NT | NT | - | - |
| 17 | Idiopathic T-cell lymphocytopenia | + | - | - | - |
| 18 | Idiopathic T-cell lymphocytopenia | - | - | - | - |
| 19 | Idiopathic T-cell lymphocytopenia | + | - | + | + |
| 20 | Multiple congenital anomalies | + | + | + | + |
| 21 | Multiple congenital anomalies | + | + | - | + |
| 22 | Multiple congenital anomalies | - | + | - | + |
| 23 | Multiple congenital anomalies | - | - | - | - |
| 24 | Multiple congenital anomalies | - | + | + | - |
| 25 | Multiple congenital anomalies | - | + | - | NT |
| 26 | Congenital diaphragmatic hernia | NT | + | + | + |
| 27 | Congenital diaphragmatic hernia | + | + | + | NT |
| 28 | Congenital diaphragmatic hernia | + | + | + | NT |
| 29 | Congenital diaphragmatic hernia | - | - | - | NT |
| 30 | Congenital diaphragmatic hernia | NT | + | - | NT |
| 31 | Congenital diaphragmatic hernia | + | + | - | NT |
| 32 | Cardiac anomalies | + | + | - | + |
| 33 | Cardiac anomalies | - | + | - | NT |
| 34 | Gastrointestinal anomalies | + | + | + | + |
| 35 | Gastrointestinal anomalies | + | - | + | + |
| 36 | Chylothorax and hydrops | + | + | + | + |
| 37 | Chylothorax and hydrops | + | + | + | + |
| 38 | Chylothorax and hydrops | + | + | + | NT |
| 39 | Sepsis and severe infections | + | + | + | + |
| 40 | Sepsis and severe infections | + | + | + | NT |
| 41 | Sepsis and severe infections | + | + | + | - |
| 42 | Sepsis and severe infections | + | + | + | - |
| 43 | Sepsis and severe infections | - | - | + | NT |
| 44 | Sepsis and severe infections | - | + | - | - |
| 45 | Sepsis and severe infections | + | - | - | - |
| 46 | Maternal immunosuppr. Use | + | + | + | + |
| 47 | Maternal immunosuppr. Use | + | + | + | + |
| 48 | Maternal immunosuppr. use | - | + | - | - |
| 49 | Maternal immunosuppr. use | - | + | - | - |
| 50 | Other neonatal condition | + | - | - | + |
| 51 | Other neonatal condition | + | + | + | - |
| 52 | Other neonatal condition | + | - | + | NT |
| 53 | Other neonatal condition | - | + | + | NT |
| 54 | Other neonatal condition | - | + | - | - |
| 55 | False-positive | - | - | + | + |
| 56 | False-positive | - | - | + | + |
| 57 | False-positive | - | - | + | + |
| 58 | False-positive | - | - | + | + |
| 59 | False-positive | - | - | + | + |
| 60 | False-positive | - | - | - | - |
| 61 | False-positive | - | - | - | - |
| 62 | False-positive | - | - | - | - |
